# Case of Fracture of Both Bones of the Left Leg, Treated in Wood’s Hammock Splint

**Published:** 1874-04

**Authors:** G. W. Bowen

**Affiliations:** Toledo, Ohio


ART. III.—*Case of Fracture of both bones of the left Leg; Treated in Woods' Hammock Splint.* By G. W. BOWEN, M. D., Toledo, Ohio.

On the 28th day of March 1873, G. R., a German laborer, aged 28 years, and of intemperate habits, while endeavoring to dismount from a loaded beer-wagon while the vehicle was in motion, had his left foot entangled in the spokes of the wheel in such a manner as to produce a transverse fracture of both bones of the leg three inches above the ankle joint. When called to see him soon after the receipt of the injury, I found him utterly oblivious to the serious nature of the injury he had sustained, learned that he had been on a spree of more than usual duration, and was now apparently on the verge of delirium tremens. On examining the leg, I found more displacement than is usual, or than I would expect in a transverse fracture. The upper end of the lower fragments were distinctly felt, apparently just beneath the skin on the inner side of the leg, which presented an ecchymosed and darkened appearance for some distance around.

Having seen the Hammock Splint of Dr. Woods of this city, used in several cases of fracture of the leg, and marked the ease with which it was borne by the patient, and the slight difficulty experienced on the part of the Surgeon to keep the broken fragments in apposition. I came to the conclusion that it would fulfill the indications in my patients' case better than any other appliance with which I was acquainted.

I accordingly procured an instrument, and with the assistance of Dr. Bond, we proceeded to adjust the fracture. After placing the limb in the Hammock, owing to the wild and restless condition of my patient, I took the precaution to pass an additional band around the pelvis and thigh, and also around the leg just below the knee; then administering a free opiate we left our patient for the night.

March 29th.—Found that my fears of the night before had been fully realized. I now had almost a compound fracture, complicated with delirium tremens to deal with—almost compound, because the injury to the soft parts had been such, that the skin presented the appearance of sloughing—which finally did occur, immediately over the point where the lower fragments were first felt. My patient had passed an exceedingly restless night, starting and attempting to arise at every little sound, and was excited by numerous imaginary visions, so much so that it had taken the constant watchfulness of at least one attendant to keep him on the bed. Notwithstanding his almost constant motion, he had not materially change the position of the broken ends of the bones.

I now placed the lower end of the splint on a firm plank, having holes bored in it so as to fasten the splint to the plank, and the plank securely to the foot of the bed. Having done this, I also passed a broad band across his chest with a view of preventing his "bobbing up" every few minutes, as he was inclined to do. At the end of about sixty hours I succeeded in procuring profound sleep, after which I had very little more trouble with the case. At the end of the fifth week I removed the splint from the leg, and supplied its place with Binders' Board dressing, and allowed my patient to sit up, and after a few days to get about with crutches.

May 16th.—Removed dressing entirely and discharged the case.

In conclusion I would state, that at this time the "provisional callus" so called, was entirely wanting, and the fragments were united so firmly and so perfectly, that the point of fracture could not be traced.

With regard to the merits of this splint as a dressing, I wish to say that I have used, and seen used the Double inclined Plane of Liston, the Fracture Box, the Plaster of Paris dressing, and others, and in my opinion the results obtained in this case, could not have been obtained without very much more trouble by any other of the many dressings heretofore in use—and I therefore would give a decided preference to the "Hammock Splint," in fractures of the lower extremity, and do not hesitate to express the belief that all Surgeons will concur with me, having once become familiar with the instrument.

